# Using an ecosystem service model to inform restoration planning: A spatially explicit oyster filtration model for Pensacola Bay, Florida

**DOI:** 10.1111/csp2.13061

**Published:** 2024-01-10

**Authors:** Philine S. E. zu Ermgassen, Jonathan R. Gair, Brandon Jarvis, Laura Geselbracht, Anne Birch, Whitney A. Scheffel, Kent Smith, Bryan DeAngelis

**Affiliations:** 1 University of Edinburgh, Changing Oceans Group, Edinburgh, UK; 2 Max Planck Institute for Gravitational Physics (Albert Einstein Institute), Potsdam, Germany; 3 US EPA, Office of Research and Development, Gulf Breeze, Florida, USA; 4 The Nature Conservancy, Florida Chapter Office, Maitland, Florida, USA; 5 Pensacola and Perdido Bays Estuary Program, Pensacola, Florida, USA; 6 Florida Fish and Wildlife Conservation Commission, Tallahassee, Florida, USA; 7 The Nature Conservancy, CA Oceans Team, Narragansett, Rhode Island, USA

**Keywords:** *Crassostrea virginica*, eastern oyster, ecosystem service, filtration, goals

## Abstract

The development of science-based restoration goals that reflect the primary motivation of stakeholders is a key factor leading to large-scale, long-term restoration successes. The ability to predict the potential ecosystem service delivery from restoration can inform the setting of appropriate goals and facilitate the strategic planning of restoration activities. While recovery of the ecosystem services provided by oyster reefs is a regularly cited reason for undertaking restoration, few examples exist where large-scale oyster habitat restoration plans have been informed using ecosystem service functions. Such an approach is currently being implemented in the Pensacola Bay System, Florida, where a broad coalition of partners and community stakeholders are utilizing a watershed approach to restoring oysters with the aim of restoring oysters for multiple objectives including habitat, ecosystem services, and wild harvest and aquaculture. Through the process of developing a habitat management plan, water filtration was identified as a key ecosystem service by the stakeholders. To support restoration planning we derived a spatially explicit estimate of water filtration services provided by the eastern oyster in the Pensacola Bay system by linking an oyster habitat suitability map to a hydrodynamic-oyster filtration model. This spatially explicit model allowed us to identify the areas where restored oyster reefs have the potential to provide the greatest increase in filtration service as well as provide spatially explicit estimates of the potential filtration provided by oyster habitat restored. Such information is useful in restoration planning and management and for stakeholder engagement, outreach, and education programs.

## 1 | INTRODUCTION

Coastal habitats have suffered significant declines globally as a result of human actions such as coastal development, pollution, and destructive fishing practices (e.g., Beck et al., 2011; Hagger et al., 2022). In response to these declines, there is a growing global movement to protect and restore habitats, as exemplified by the UN decade on restoration (United Nations General Assembly, 2020a, 2020b). While there may be many diverse motivations to restore habitats, the recovery of ecosystem services is often cited as a key driver (Bayraktarov et al., 2020; Hagger et al., 2017). Yet many restoration projects are implemented with minimal acknowledgement or understanding of how an individual restoration project contributes to ecosystem-scale (e.g., bay or estuary-wide), functional or regional management goals (Hobbs & Harris, 2001). As such, the development of science-based, estuary-level goals, has been identified as a key factor leading to landscape—scale restoration successes in the past (DeAngelis et al., 2020). It is important that the identified goals reflect the primary motivation for the restoration efforts, as identified by what the community is trying to achieve through restoration (e.g., increased recreational opportunities, increased wildlife, improved ecological condition; DeAngelis et al., 2020, Zu Ermgassen et al., 2016). Predicting the ecological outcomes of restoration activities as they pertain to the identified goals is an important step to allow strategic planning for optimizing the delivery of the restoration goals and to feed into adaptive management of the sites (Hobbs & Harris, 2001).

The Pensacola Bay System (PBS), defined here as Pensacola, Escambia, East, and Blackwater bays, is the fourth largest estuary in the state of Florida, USA. The PBS once supported a robust eastern oyster (*Crassostrea virginica*) population and associated oyster fishery (McNulty et al., 1972), with extensive oyster beds historically occurring throughout the PBS (Lewis et al., 2016; McNulty et al., 1972). Oyster die-offs were reported in the 1950s and attributed to poor management of the fishery and habitat, disease, poor water quality, sedimentation, and a lack of suitable substrate for settlement (Collard, 1991; WFRPC, 2005; Lewis et al., 2016). Comparison with the extent mapped in the late 1800s, however, suggests that significant declines predate the 1950s (US Fish Commission 1883, cited in Birch et al., 2021). Recent mapping efforts have highlighted the degraded status of the remaining oyster reef areas in the PBS, with the vast majority of remaining oyster areas supporting negligible densities of oysters (Johnson, 2021a,b). This recognized habitat decline and the collapse of the oyster fishery have resulted in a desire in recent years to restore oyster reef habitats to the PBS (Birch et al., 2021).

In the absence of a comprehensive oyster management and restoration plan, the State of Florida and a diverse consortium of stakeholders championed the development of a bay-specific plan to provide a path to recovery of oysters, entitled *Oyster Fisheries and Habitat Management Plan for the Pensacola Bay System* (PBS Oyster Plan) (Birch et al., 2021). The PBS Oyster Plan recognizes the oyster fishery, aquaculture industry, and habitat created by oysters as equal elements in development of the goals and strategies for restoration and management. Proposed management actions are aimed at achieving ecological outcomes and social objectives, in addition to increasing oyster fishery production. The result is a model for community ownership and management based on the best available science.

The PBS Oyster Plan laid out a set of strategies to recover the oyster population, along with a series of associated actions that should be undertaken to complete the identified strategies. These strategies were subsequently ranked in order of priority by the participating stakeholders, which included oyster harvesters and oyster aquaculture farmers, state and local government agencies, Pensacola and Perdido Bays Estuary Program, businesses, universities, and community members. One of the strategies identified as among the most important, being to “establish restoration and management targets for functional harvested and non-harvested oyster reefs using 1–3 ecological health indicators.” Two actions associated with that strategy involved creating a list of priority restoration projects for the bay system based on restoration and management targets; and to establish ecosystem targets to manage the bay system (Birch et al., 2021). As a continuation of this planning effort, the Pensacola and Perdido Bays Estuary Program (PPBEP) committed to integrating the top priorities identified in the PBS Oyster Plan into their first Comprehensive Conservation and Management Plan (CCMP), a blueprint for the long-term protection and recovery of the Pensacola and Perdido Bay watersheds (Pensacola & Perdido Bays Estuary Program, 2022; https://www.ppbep.org). The strategies and actions outlined by the stakeholders of the PBS and the PPBEB CCMP (PPBEB, 2022) reflect a desire to set ecosystem level goals for oyster habitat restoration. Critically, discussions with stakeholders identified an initial oyster reef restoration goal of 600 ha, which was based on the extent of oyster reefs that were mapped prior to the 2010 Deepwater Horizon oil spill in the Gulf of Mexico (Goal 5 in Pensacola & Perdido Bays Estuary Program, 2022).

Stakeholders involved in the PBS Oyster Plan deemed water filtration by oyster habitats to be one of the key ecological services to base restoration and management targets around (Birch et al., 2021; Pensacola & Perdido Bays Estuary Program, 2022). Stakeholders generally valued the role that filtration may play in improving water clarity, which in turn was valued for esthetic and recreational purposes. Oysters are suspension feeders whose filtration reduces phytoplankton and suspended sediment particles >5 μm in size with high efficiency (Riisgaard, 1988), thereby improving water clarity.

Filtration services underpin a range of processes that support improved water quality. Filtration by oysters and wave attenuation by shallow oyster reefs can have a positive impact on light penetration and hence seagrass growth and recovery (Newell & Koch, 2004; Smith et al., 2009; Wall et al., 2008; Wall et al., 2011), while filtration by oysters can also increase the availability of nutrients in the sediments and result in increased seagrass growth rates (Booth & Heck Jr., 2009). Furthermore, the consumption of algae and other organic matter from the water column through filtration contributes to the removal of nutrients, including nitrogen and phosphorous from the water. This removal of nutrients arises both via assimilation of the nutrients into the oyster’s tissue and shell (Kellogg et al., 2013), and via oysters enhancing nitrogen removal by creating conditions conducive to denitrification and burial of organic matter (Newell et al., 2005). Although the impact of filtration on these ecosystem benefits is spatially variable and the relationship between oyster density and such benefits remains poorly parameterized (Booth & Heck Jr., 2009; Newell & Koch, 2004), there is empirical evidence that these benefits scale with oyster density and extent (Smyth et al., 2015).

Here, we develop a model for estimating the potential filtration service by restored oyster reefs in PBS. Filtration service, as predicted by the model presented here, can be used as a simple proxy for communicating the many complex and critical ecological benefits arising from filtration by oyster reefs, to inform stakeholders and support in developing oyster restoration and management targets. The approach developed here is similar to that developed by Gray et al. (2019), in which the filtration service provided by *Ostrea lurida* in Yaqunia bay, Oregon was estimated across its historical distribution based on existing abiotic conditions and a hydrodynamic model of the estuary.

In the case of the PBS, the historical extent of oyster reefs was not accurately mapped, although a rough map drawn up in the 1880s indicated that oyster reefs were expansive and scattered throughout the PBS (US Fish Commission, 1883, cited in Birch et al. 2021). A bay-level restoration scenario was therefore developed, based on a habitat suitability model for subtidal oyster reefs (Geselbracht et al., n.d.), and existing mapping of more recent past oyster reef extent. Our aim was to identify the areas within the bay where restoration would provide the greatest filtration service, both as restoration progresses (order of restoration), and once large-scale restoration (1978 ha restored) was complete. By virtually placing oysters throughout the estuary in areas of high and medium habitat suitability, we identified locations (grid cells) that are likely to provide the greatest filtration service, expressed as a proportion of particles in the bay removed by oysters within each cell, should restoration be realized. Additionally, the model generated a rank order of cells, identifying which cell contributed the greatest increase in filtration services consecutively, and an estimate of the volume of water cleared of particles at each individual location. Such values can be used as an important decision making and communication tool when working toward long-term, large-scale goals that can take decades to accomplish (Druschke & Hychka, 2015). The results of this project are already being used in tandem with the habitat suitability model and stakeholder engagement to support decision making for recovery, restoration, and management goals for the eastern oyster in the PBS.

## 2 | METHODS

### 2.1 | Hydrodynamic model

**FIGURE 1:**
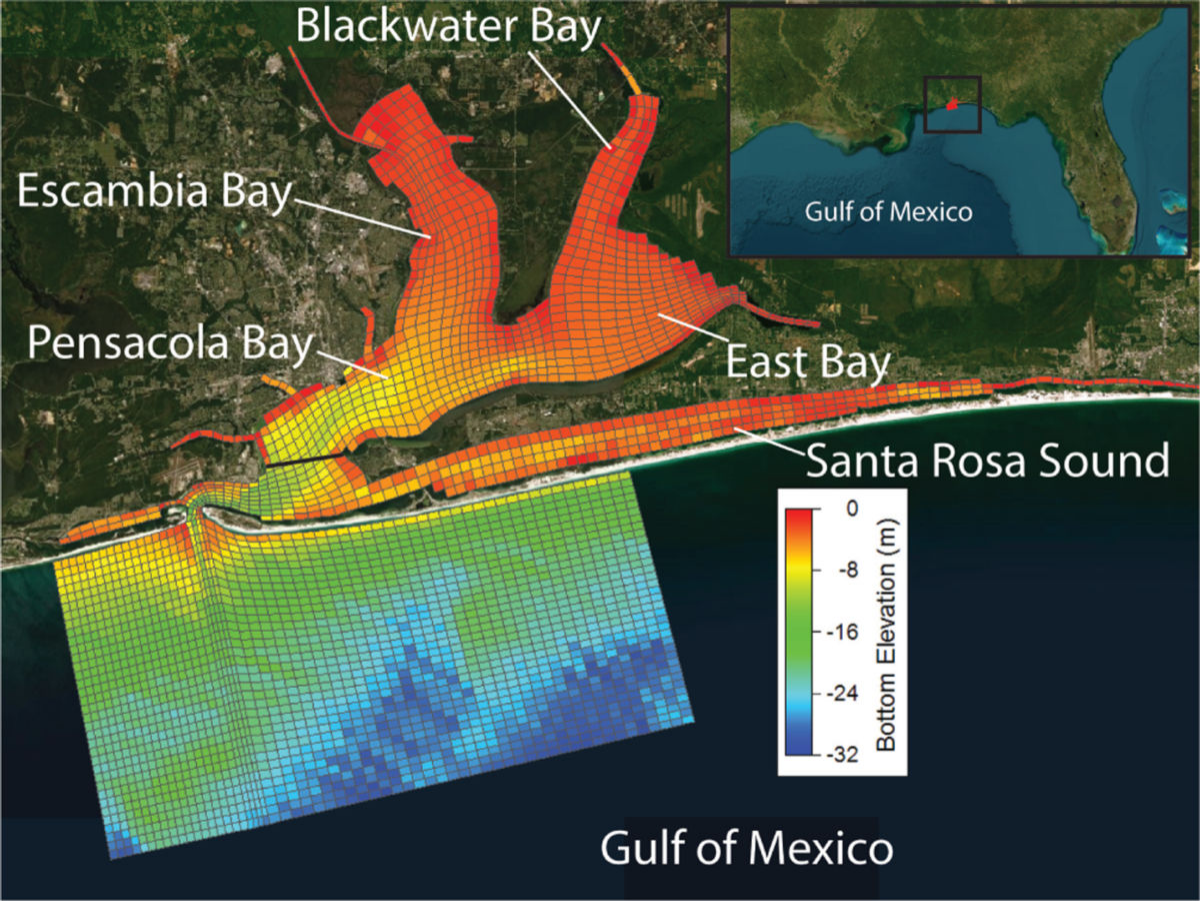
Map of the Pensacola Bay System, Florida including the EFDC hydrodynamic model domain. The black line south of Pensacola Bay depicts the southern boundary of the oyster filtration model. Bottom elevation in Mean Lower Low Water (m) from National Oceanic and Atmospheric Administration (NOAA) (2022) is provided for context of model output and oyster habitat suitability.

Spatially explicit water temperature and salinity estimates and hydrodynamic forcings for the oyster filtration model were provided by a three-dimensional simulation model of PBS developed using the Environmental Fluid Dynamics Code (EFDC) previously described by Duvall et al. (2022). The hydrological model accounts for the estuary receiving freshwater inputs from three main rivers: Escambia, Blackwater, and Yellow Rivers, and models water movements throughout five interconnected waterbodies including Pensacola Bay, Escambia Bay, Blackwater Bay, East Bay, and Santa Rosa Sound, and south of Pensacola Pass in the Gulf of Mexico (Figure 1). The PBS has an average depth of 6 m, with the bottom sediments predominantly made up of silts and sands (Schroeder & Wiseman, 1999). Low amplitude diurnal tides range between 0.15 and 0.65 m (mean: 0.37 m) (National Oceanic & Atmospheric Administration (NOAA), 2022).

EFDC is a state-of-the-art hydrodynamic model based on turbulence-averaged governing equations and is suitable for application in freshwater and coastal ecosystems, including tidal estuaries. Solution techniques for the equations in EFDC are presented in Hamrick (1992, 1996) and summarized in Hamrick and Wu (1997). The EFDC model grid was developed using the CVL grid generator (DSI international; https://www.eemodelingsystem.com/), and includes 3299 horizontal grid cells. The model domain covers 1162 km^2^, with an average horizontal grid area of 0.36 km^2^ and minimum and maximum horizontal cell face dimensions ranging between 118 and 1428 m, respectively. Hydrodynamic and particle tracking simulations were performed using DSI’s EFDC Explorer V10.2 software (DSI international; https://www.eemodelingsystem.com/). Simulations included Lagrangian particle tracking utilizing 2000 particles established at random horizontal positions throughout the Central PBS (excluding areas south of Pensacola Pass in the Gulf of Mexico).

Hydrodynamic transport in the hydrodynamic model is controlled by tides, river discharge, and wind, the effects of which vary spatially and temporally (Hagy III & Murrell, 2007). We estimated the open water tidal boundary and wind forcing using the National Oceanic and Atmospheric Administration (NOAA) PCLF1 monitoring station, with a constant salinity (36 ppt) and measured temperature from NOAA buoy 42,012. Atmospheric forcing, including atmospheric pressure, air temperature, relative humidity, precipitation, cloud cover, and solar radiation, were provided by the European Centre for Medium-Range Weather Forecasts (ECMWF) ERA5 atmospheric reanalysis of the global climate (Copernicus Climate Change Service (C3S), 2020). Major freshwater river discharges were estimated using four United States Geologic Survey (USGS) gage stations. Smaller rivers and permitted discharges to the estuary were estimated by scaling the Escambia River and Yellow River discharge measurements and applying permitted discharge limits, respectively.

Model calibration was performed using a one-year simulation in 2016, and further validation was performed using a one-year simulation in 2014. The 2014 and 2016 calibration/validation years represent years with relatively low and high spring discharge, respectively, and were selected to evaluate the model’s accuracy in addressing varying flow conditions with a single calibration. Calibration and validation of water surface elevations, temperature, and salinity were performed using NOAA observational data and continuous monitoring data collected at the surface and bottom layer at four monitoring stations in Pensacola Bay (Figures S1–S6).

We chose to run the hydrodynamic model starting May 1st to represent the period of enhanced spring discharge, which aligns with increasing phytoplankton growth and primary production in Pensacola Bay resulting from increasing water temperatures and high nutrient loads (Murrell et al., 2018). Enhanced freshwater discharge further results in broad distribution of the freshwater plume across portions of the mid-bay during periods of moderate to high flows (Hagy III & Murrell, 2007). While deeper portions of PBS are known to intermittently stratify for long periods of the spring–summer (Hagy III & Murrell, 2007), shallower regions outside the main channel are highly susceptible to wind and tidal mixing on subdaily timescales, and may therefore be considered mixed (Duvall et al., 2022). It is these shallower areas in which the high and medium areas of habitat suitability for oyster reefs are predominantly located (Figure S7).

### 2.2 | Oyster filtration model selection and factors

The rate at which particles are cleared from the water is commonly termed the clearance rate (CR). Zu Ermgassen et al. (2013) developed a CR model based on field data collected upstream and downstream of oyster reefs (Grizzle et al., 2006; Grizzle et al., 2008) and determined the CR of eastern oysters *in situ* under optimal temperature conditions to be equivalent to 8.02*W*^0.58^, where W is the dry tissue weight of the oyster in grams. This “optimum” clearance rate is reduced under suboptimal temperature, salinity and dissolved oxygen conditions (Cerco & Noel, 2005). The *in situ* model derived by Zu Ermgassen et al. (2013) has also been used by others to demonstrate nitrogen removal services provided by oysters in the northern Gulf of Mexico (Lai et al., 2020). Data from PBS for abiotic factors affecting CR were identified from literature (Table 1). Within the spatially explicit model, it was possible to incorporate salinity (S) and water temperature (T) (Equation 1).


(1) $$CR=8.02W^{0.58}e^{-0.015{\left(T-27\right)}^{2}}\times (0.5(1+\tanh(S-7.5)))$$

### 2.3 | Habitat suitability model

A habitat suitability model was developed for oyster habitats in the PBS (Geselbracht et al., n.d.). Factors included in the model were: areas identified as oyster reef for the 1995–1997 Environmental Sensitivity Index and 2010 data from the Florida Department of Agriculture and Consumer Services (FDACS) (Radabaugh et al., 2019) (available as publicly accessible GIS datasets https://geodata.myfwc.com/datasets; Accessed November 13, 2019), historical oyster reef (1883), bottom dissolved oxygen, seagrass/vegetation presence/absence, sediment type, salinity, and oyster recruitment (see Table S1 for data sources and details of the respective weightings applied). The model assigns a habitat suitability score of low, medium, or high for areas of the PBS. In this modeling effort, only areas identified as having high or medium habitat suitability were included when mapping potential areas of restored oyster reef and hence for estimating potential filtration services.

#### 2.3.1 | Modeled extent of potential restored reef

The habitat suitability model identifies broad regions of the PBS as suitable for oyster habitat restoration (Figure S7), yet as oyster reefs are patchy by nature (Kasoar et al., 2015), continuous coverage of suitable areas are highly unlikely. To estimate the average proportion of these broad areas that may reasonably support oyster reef formation, we determined what proportion of each contained oyster reef in previous mapping efforts. As in the habitat suitability model, we used oyster reef extent from the publicly available mapping data between1995 and 2010 (https://geodata.myfwc.com/datasets; Accessed November 13, 2019). Within each grid cell as delineated in the EFDC hydrodynamic model (Figure 1) we determined by least squares fit the proportion of each habitat suitability model category (medium or high habitat suitability; Figure S7) identified as oyster reef in the GIS oyster layer. Cells in which oysters were absent in the GIS data were not included in this calculation. The projected restorable area within each cell was estimated by applying the least squares fit derived mean proportion of oyster coverage, to the extent of high and/or medium habitat suitability in all cells. Only grid cells north of the Santa Rose Sound/Pensacola Bay dividing line (see Figure 1) were included in the analysis.

#### 2.3.2 | Developing a reef-level oyster density and size class restoration scenario for PBS

Filtration services scale with the size and abundance of oysters (Cranford et al., 2011), yet there is a lack of long-term data on oyster density and size class on successfully restored oyster reefs in PBS. We therefore first needed to establish reasonable reef-level oyster demographic parameters that are representative of a “successful” restoration effort in the northern Gulf of Mexico. Deriving a potential oyster restoration scenario provides additional opportunities, both to communicate what can be anticipated from successful restoration with regards to ecosystem service provision, and to consider how such information can be incorporated into management of the resource, for example, through goal setting (Daily et al., 2009; Guerry et al., 2015).

In the absence of long-term restoration monitoring data, we developed a scenario of mean oyster size and density based on the guidelines outlined by the Oyster Metrics Workgroup (OMW) in the Chesapeake Bay (Oyster Metrics Workgroup, 2011). The Oyster Metrics Workgroup developed a series of recommendations for assessing the success of oyster restoration efforts in the Chesapeake Bay. Their recommended operational success criteria were 50 oysters m^−2^ and 50 g DTW m^−2^, covering at least 30% of the reef area, thus averaging as 15 oysters m^2^ over the oyster reef. We applied this average threshold density for “successful” oyster restoration criteria, to data from the scientific literature on unfished natural and restored oyster reef sites in the northern Gulf of Mexico (La Peyre, Furlong, et al., 2014; La Peyre, Humphries, et al., 2014; Nevins et al., 2014).

Oyster density and size class data were extracted from three key studies in the northern Gulf of Mexico, each representing different oyster reef attributes (La Peyre, Furlong, et al., 2014; La Peyre, Humphries, et al., 2014 and Nevins et al., 2014). The mean density of oysters that might be expected in a “successful” restoration scenario was derived from La Peyre, Furlong, et al. (2014). La Peyre, Furlong, et al. (2014) provides a comprehensive review of oyster restoration efforts in the northern Gulf of Mexico. The mean density scenario was derived by extracting the presented field data from restoration sites that achieved successful restoration as defined by Oyster Metrics Workgroup (2011) (more than 15 oysters m^−2^). The proportion of those oysters that represented spat (<25 mm shell height [SH]), seed (25–75 mm) and market (>75 mm SH) oysters was similarly estimated based on the data presented in La Peyre, Furlong, et al. (2014). Data from projects using shell substrates were excluded, as this substrate predominantly represented restoration efforts that did not achieve densities reflective of “successful” restoration. Mean SH of spat oysters was extracted from 3 year old restored reefs reported in La Peyre, Humphries, et al. (2014), while the mean SH for the market and seed oysters categories were derived from Nevins et al. (2014). Most available monitoring for oyster reefs are either from fished reefs, or reefs that have been recently restored and that are therefore likely to be represented by a truncated size distribution. Nevins et al. (2014) presents SH from samples on unfished oyster reef in Sabine Lake, Texas, which allows for representation of a full size distribution as might be expected on an unfished reef in the long term. Nevins et al. (2014) was not used to estimate spat SH, as dredges are known to sample smaller oysters less efficiently (Marenghi et al., 2017), and was therefore deemed less suitable for deriving data in the smallest size class.

#### 2.3.3 | Shell height to dry weight conversion

Oyster CR is usually reported relative to oyster dry tissue weight (DTW). Dry tissue weight varies by growth, location, and time of year. It is therefore recommended that SH-weight conversions be taken from nearby sites with similar characteristics if they do not exist for the area of interest. We were unable to identify SH-weight conversions for PBS, nor for similar sites nearby. Data sampled by Edwards (2014) in Grand Bay, Mississippi Sound (110 km west of PBS) provided the nearest identifiable SH to DTW conversion from wild reefs (Equation 2). It should be noted that there are differences between Grand Bay and the PBS: while both estuaries exhibit low amplitude diurnal tides, Grand Bay exhibits generally higher salinities (Point Aux Chenes NOAA NERR monitoring site = ~22 ppt) than Pensacola Bay (central bay site P5 depth averaged salinity = 17 ppt) and may be considered more of a marine system. Nevertheless, no better analogous site could be identified from the literature.

(2) $$DTW(\text{g})=0.0001^{*}L^{2.1}$$

where L = shell height in mm.

#### 2.3.4 | Deriving spatially explicit models of filtration

Filtration service refers to the total reduction of particles resulting from modeled oyster CR throughout the bay. The particle tracking data obtained from the hydrodynamic model, as well as the model-derived salinity and temperature in each cell, was used to determine the filtration service that could be provided by restoration of oysters within PBS over a 2-week time period starting 1 May 2015. The hydrodynamic model tracks the locations of 2000 particles released at the start of the model run. Code was developed in C++ to apply cell-specific oyster CR at each time step. The duration of a time step was 1 h. Clearance rates were computed assuming the estimated proportion of high and medium habitat suitability areas within each hydrodynamic cell was successfully restored.

The filtration service was computed by assigning each released particle a starting value (or “load”) of one, which was then reduced in each time step if the particle was in a cell modeled to contain oysters. At each time step, the load was reduced by a factor of exp(−FT), where F is the filtration rate of the cell in which the particle was located at that time step, and T is the time interval (1 h). The total filtration provided by a given cell was computed as the decrease in load summed across all particles at the end of the whole simulation period (i.e., 2 weeks). The modeled impact of filtration by oysters assumes that the water is perfectly mixed, that is, that the load on the particle is instantly distributed uniformly over the cell volume at the start of the time step.

In a separate model run, the rank order in which cells might be prioritized for restoration was determined. In this case, the model started by assuming that all cells were empty of oysters, and then identified stepwise which grid cells identified as containing habitat suitable for oyster restoration would add the greatest filtration service, until all grid cells containing high or medium habitat suitability areas were “restored.” The best cell to restore at each step was defined as the cell whose inclusion would give the greatest increase in the filtration service, given the cells already previously restored.

## 3 | RESULTS

### 3.1 | Oyster parameters

We determined that the average proportion of a given area of high and medium suitability habitat containing oyster reef in the 1995–2010 mapping data was 0.177 and 0.110, respectively. Therefore 17.7% of all areas of high habitat suitability and 11% of all areas of medium habitat suitability were projected to be restorable. The resulting area of projected restorable oyster reef was 1978 ha across the whole PBS. While this projected extent is significantly greater than the extent of oyster reef represented in any recent assessment of the Bay (Radabaugh et al., 2019), this represents only 58% of the area mapped in 1972 (3394 ha, McNulty et al., 1972, cited in Lewis et al., 2016) and only approximately 13% of the area delineated in a chart dating from 1883 (15,000 ha; US Fish Commission 1883 cited in Birch et al., 2021).

The mean density of oysters at all reefs surveyed in La Peyre, Furlong, et al. (2014) that met the criteria of supporting densities >15 m^−2^, was 221 oysters m^−2^, which was adopted as the density for our “successful” restoration scenario. When the relative proportion of oysters in each of three size classes was applied, this resulted in a density of 61 oysters m^−2^ for spat (<25 mm), 125 oysters m^−2^ for seed (25–75 mm) oysters, and a density of 34 oysters m^−2^ for market oysters (>75 mm). Predicted mean size within each size class was 16, 56, and 98 mm SH, respectively (Table 2).

### 3.2 | Spatially explicit oyster filtration

**FIGURE 2:**
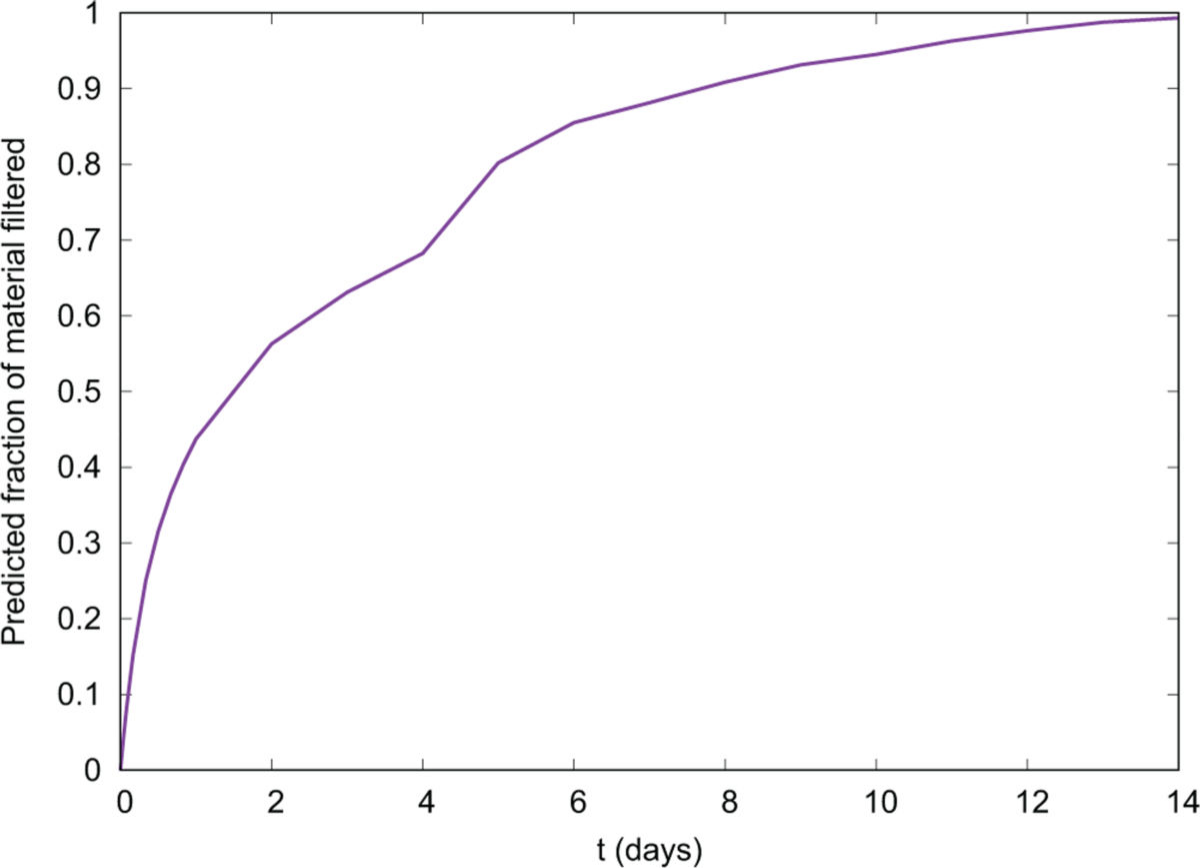
The fraction of particles in the hydrodynamic model which were cleared from the water column over time once high habitat suitability and medium habitat suitability areas were restored under a “successful” restoration scenario (>15 oysters m^−2^). The proportion of high and medium habitat suitability areas estimated to be covered by oyster reef in this scenario was determined by least fit squares to oyster reef mapped from 1995 to 2010. See methods for details. “Full estuary filtration” was achieved in ~14 days.

**FIGURE 3:**
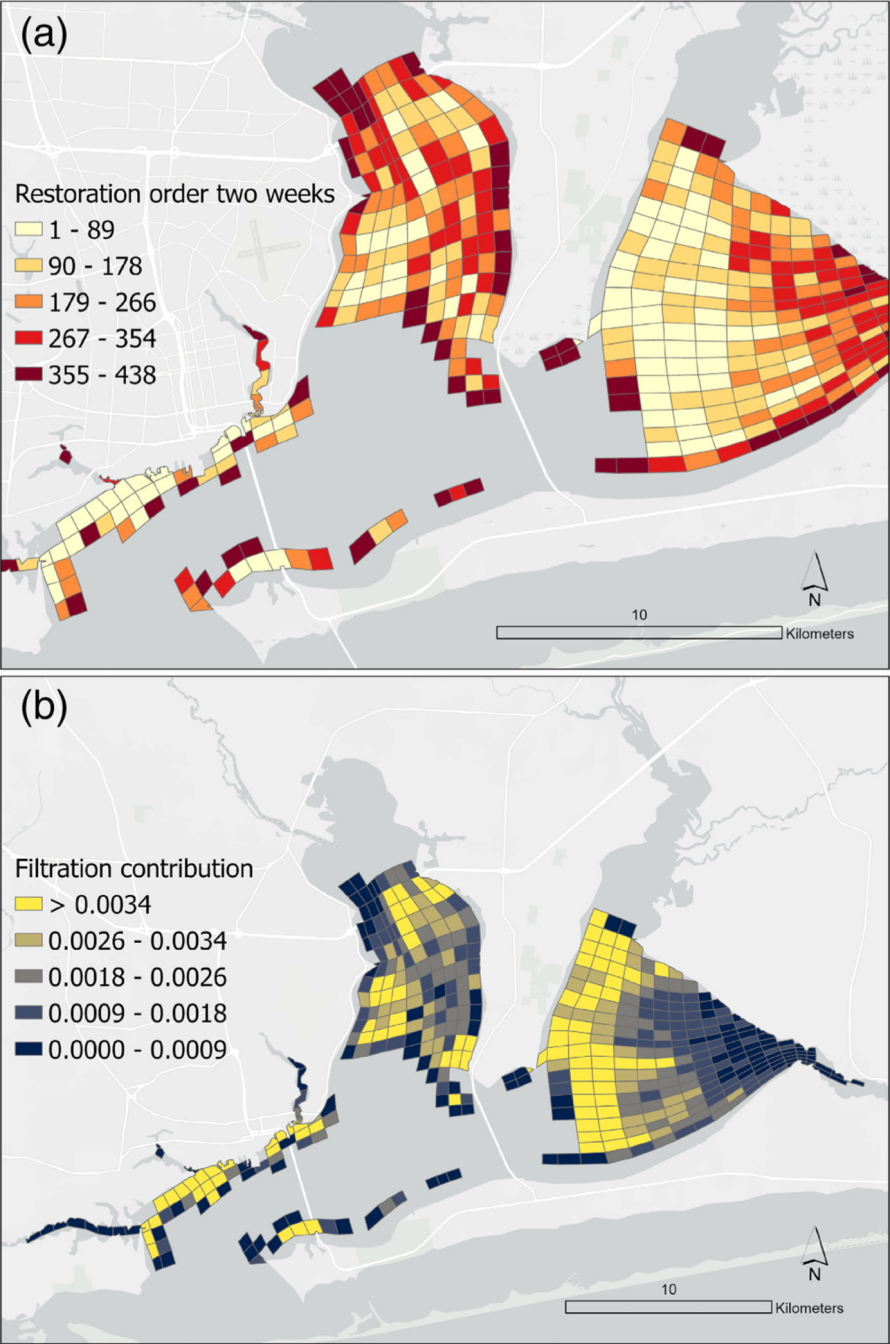
Maps illustrating (a) restoration order and (b) filtration contribution of cells under a successful restoration scenario, 2-week model run. Restoration order indicates which cells contribute the greatest additional increase in filtration when restored sequentially. Filtration contribution illustrates the proportion of modeled particles removed each individual cell over the 2 week model run.

Our spatially explicit model of oyster filtration in a restored PBS indicated that restoration of oyster reef has the potential to provide a significant filtration service. Full estuary filtration was predicted to be achieved in ~14 days, turning over the full volume of the estuary approximately twice within the 27-day residence time of water in the system (Bricker et al., 2007, see Figure 2). Several model outputs were generated: (i) the restoration order of each cell, which is the cell whose inclusion provides the greatest filtration service, while accounting for the cells already previously restored (Table S2, Figure 3a); (ii) the filtration contribution provided by each cell, which is measured as the decrease in load summed across all particles at the end of the whole simulation period (Table S2, Figure 3b); (iii) the average filtration rate of each cell, measured in m^3^ h^−1^ (Table S2); and (iv) the filtration in L h^−1^ m^−2^ of oyster habitat, in each cell (Table S2).

**FIGURE 4:**
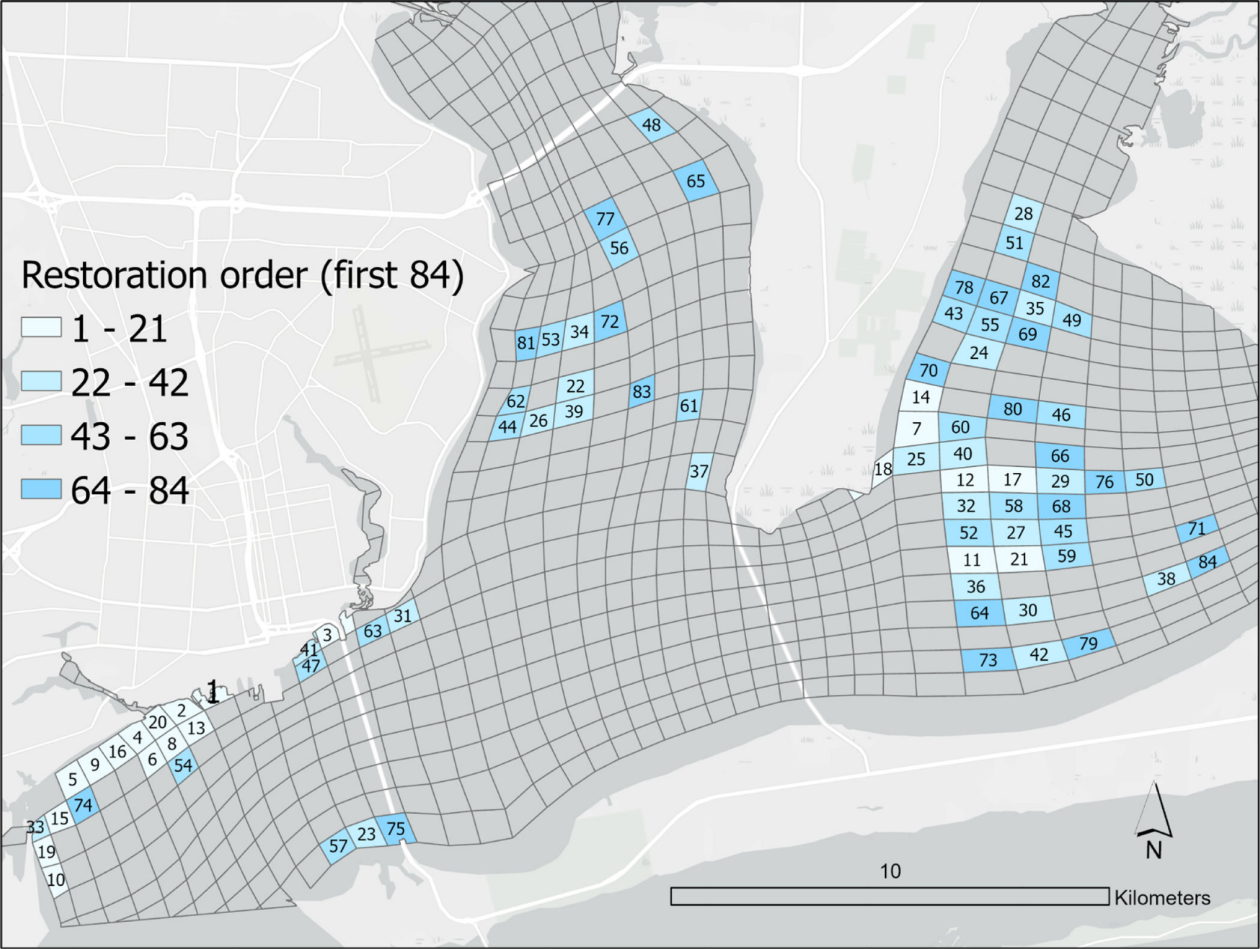
Restoration order of the first 84 cells, which would provide the greatest additional filtration if restored sequentially, starting with grid cell 1. The 84 cells represent ~600 ha of potential restored oyster reef area. This area is equivalent to that agreed by stakeholders as an initial oyster reef restoration goal (Goal 5 in Pensacola & Perdido Bays Estuary Program (2022)).

The model identified the northern shore near the entrance to Pensacola Bay as an area where several grid cells were predicted to provide the greatest filtration contribution (Figure 3a) and were ranked highest in proposed restoration order to maximize filtration services (Figures 3b and 4). The western portion of East Bay was also predicted to contribute disproportionally to filtration services in the PBS. It is notable that some of the top ranked cells in restoration order, were modeled to contain relatively small extents of potential restored reef (e.g., the top ranked cell in restoration order (Figure 4), was projected to contain only 2.3 ha restored reef (Table S2). While filtration rates in that cell were predicted to be high (264 L h-1 m^−2^ oyster reef), they were by no means the highest (e.g., the cell ranked 34 in Restoration Order was predicted to support a filtration rate of 387 L h^−1^ m^−2^ oyster reef), indicating the important role of water movements in dictating where the greatest filtration services may be delivered.

## 4 | DISCUSSION

The goal of this study was to help inform decision making related to specific strategies and actions that were developed by a large consortium of local and regional stakeholders to advance recovery of oysters in the PBS (Birch et al., 2021; Pensacola & Perdido Bays Estuary Program, 2022). By combining an oyster habitat suitability model, a hydrodynamic model of the system and a model of oyster particle clearance rates, we were able to generate several predicted outputs that can inform bay-level restoration goals and site selection, which are subsequently being used in PPBEP led efforts to engage stakeholders. Visualizations of the order of ranked cells up to a restoration area of ~600 ha (the interim reef restoration goal identified in Goal 5 of the PPBEP, 2022), such as illustrated in Figure 4, assist in the prioritization of potential restoration sites and when engaging with stakeholders. The model also allows for estimation of the volume of water cleared within each of the cells given a “successful” restoration scenario (Table S2), which again can be used both for stakeholder engagement and for decision making.

In the approach used, the relative importance of any cell in the PBS in clearing particles is a function of several factors, including: the underlying abiotic conditions (i.e., S and T); the direction and speed of movement of particles around the PBS; the area within each cell suitable for oyster restoration predicted to be restored; and the density and sizes of oysters restored in the cells. Oysters “upstream” of any cell may already have reduced the available particles in the model. There is therefore not a perfect correlation between filtration contribution once the full restorable area of the bay is restored and the order in which it is recommended that cells be restored, nor the estimated rate of filtration from a given m^2^ of oyster habitat. Stakeholders and practitioners may decide to prioritize either the modeled contribution once the full area of possible oyster reef is restored (filtration contribution in Table S2), or the step-wise contribution (rank order in Table S2, Figure 4).

In addition to being used in project planning, the model outputs can also be used to help communicate the benefit of implementing individual restoration projects by quantifying the localized filtration rates in the context of large-scale (and longer-term) restoration efforts, as well as to illustrate the potential long-term, large-scale impact of restoration on the delivery of a key ecosystem service. In this case, the model illustrated that if 1978 ha of oyster reefs were “successfully” restored to the PBS, they would have the potential to clear the entire estuary of particles in ~2 weeks, turning over the full volume of the estuary approximately twice within the 27-day residence time of water in the system (Figure 2). Implementing restoration at the bay level will involve multiple years of effort, accumulated over numerous smaller projects. Being able to quantify and communicate how each new built project is contributing individually, as well as its contribution toward the system-wide goals, can serve as a powerful communication tool over this long duration.

The approach outlined here can be applied to any estuary for which hydrological models have been developed (e.g., Gray et al., 2019, 2022). Several model assumptions should, however, be considered when determining whether application of this model is appropriate. Our model assumes complete hydrodynamic mixing within cells, which is likely to be the case in the shallow-water locations of the PBS but may not hold true in deeper water (Duvall et al., 2022). While we considered this assumption to be reasonable given the spatial distribution of the areas of high and medium habitat suitability for oysters in the shallower regions in the PBS, this is not the case throughout the PBS. The validity of this assumption should also be contemplated when applying this model to new locations. A second assumption in our model, was that the oyster population does not currently contribute significantly to filtration services. This assumption allowed us to run the model placing oysters “from scratch,” essentially ignoring the contribution of existing oysters in the bay. We considered this a reasonable starting assumption given the currently degraded state of oyster habitats in PBS (Johnson, 2021a,b), but as oysters recover, the existing population may also be accounted for in the starting conditions. The appropriate starting conditions should also be considered when applying this model to new locations. Additionally, the model can be run over differing seasons or durations, depending on the dynamics of the estuary to which it is applied, or the aims of the oyster restoration effort. We chose to run the model to coincide with spring phytoplankton blooms, and peak river discharge into the PBS. The duration most appropriate to assess the impact of oyster filtration will also vary, depending on the residence time of the bay and the aims of the restoration effort (in this case to improve water clarity at a large scale). A 2-week model run was selected under this restoration scenario in the PBS, because this represented the point at which all particles were removed and fell well within the 27-day residence time of water in the system. This run duration not only provides a basis for communicating that if oysters were restored to the PBS, they would have the potential to provide a significant filtration service, but also allows the relative contribution of cells to be assessed over an ecologically relevant time period.

Our “successful” restoration scenario was derived based on field studies on unharvested reefs (La Peyre, Furlong, et al., 2014; La Peyre, Humphries, et al., 2014; Nevins et al., 2014), as the PBS Oyster Plan highlights the importance of restoring reefs for their ecological function and services (Birch et al., 2021), which is best achieved through protection (Grabowski et al., 2012). The resulting oyster densities in our “successful” restoration scenario are modestly higher than the lower limit of what must be achieved in order to support “Healthy oyster reefs capable of sustaining commercial harvest” as defined by the FDACS’ Standard Oyster Resource Management Protocol (cited in Florida Department of Environmental Protection, 2022). Reefs are considered “Healthy oyster reefs capable of sustaining commercial harvest” when they have more than 400 bags of oysters per acre, which is equivalent to roughly 22 market oysters m^−2^, whereas our “successful” restoration scenario assumed 34 oyster m^−2^ (Table 2). Our “successful” scenario densities are within the range of what was recorded at some NDRA restoration sites in the western portion of the PBS (Florida Department of Environmental Protection, 2022), although those densities have yet to be sustained the long term (Florida Department of Environmental Protection, 2022). Nevertheless, our scenario densities appear biologically plausible, should recovery be achieved in the PBS.

In practice, the PBS Oyster Plan seeks to restore a mosaic of oyster reef management priorities, including wild harvest reefs and living shoreline reefs, in addition to protected areas of subtidal oyster reef. Consideration of the impact of fishing on the delivery of filtration services, as well as consideration of the density and extent of current or recently restored oyster habitats could be incorporated in further model runs, as subsequent rounds of stakeholder engagement take place and decisions regarding restoration areas and goals are reached. Similarly, practitioners using this approach in other areas may choose to set different oyster demographic descriptors, based on the likely management and underlying available oyster demographic data available for their location.

The “successful” restoration scenario applied in the reported model run was based on the mean density of all restoration sites sampled from unfished natural and restored oyster reef sites in the northern Gulf of Mexico that achieved more than 15 oysters m^−2^. While not reported in this study, we also developed a scenario and associated modeled outputs using a “highly successful” restoration scenario of sites which achieved more than 50 oysters m^−2^ following restoration (Table S3). The relative contribution of cells did not differ substantially between the “successful” restoration scenario and the “highly successful” restoration scenario (Figures 3 and 4; Figures S8–S10). The spatial consistency of the two model runs, despite the differing oyster biomasses represented, lends credence to the recommendations arising from the model results.

Across the United States, oyster habitat is largely managed as a fishery rather than both as a fishery *and* a non-extractive habitat, despite the fact that oysters could yield greater ecosystem services, including those supporting local and regional economies, through the non-extractive benefits they provide (Grabowski & Peterson, 2007). The focus on managing oyster habitats for extraction, has a centuries long history (Kirby, 2004), but has done little to slow the dramatic declines in oyster extent and density (zu Ermgassen et al., 2012). While there are now a growing number of success stories of using non-extractive ecosystem services to inform management (e.g., Ruckelshaus et al., 2015), management of oyster habitats that accounts for the broad array of services they provide is still not an approach typically attempted for managing the oyster resource. Aptly, the PBS Oyster Plan focuses on restoring and managing oysters for multiple functions and services, including recreational fisheries, shoreline and adjacent habitat protection, and others (Birch et al., 2021), with the model outputs presented here providing part of the evidence presented to stakeholders during the restoration decision making process (PPBEP Pers. Comm.).

Setting large-scale, ambitious, yet meaningful goals that a group of stakeholders and the community can support and identify with is one of the most important elements of long-term, landscape-scale restoration success (DeAngelis et al., 2020). Similarly, a clear set of performance metrics are critical to inform goals for restoration implementation and adaptive management (Baggett et al., 2015; Fitzsimmons et al., 2020). Given the scale of the recent decline in the PBS oyster population (Camp et al., 2015; Radabaugh et al., 2019), there is great interest within the community to make substantial inroads into recovering the oyster population, and a lack of information to guide the setting of performance metrics. The extent of restoration required to recover filtration services can be considered as one factor to consider when setting long term goals. Both the projected restorable area and the field-derived oyster density and size class scenarios developed through this effort can be used to inform goal setting and the setting of performance metrics.

Many factors need to be accounted for in site selection for restoration, including biotic and abiotic factors, as well as the socioeconomic setting, and logistical factors (Fitzsimmons et al., 2020; Hughes et al., 2023). The potential for a given site to contribute to the stakeholder identified goals of restoration should also be considered. By predicting the potential contribution of the projected restorable oyster reef area in all high and medium habitat suitability areas of the PBS (totaling 1978 ha), these model outputs can serve to inform site selection for the initial 10-year areal extent goal for oyster restoration (set at 600 ha by the CCMP; Pensacola & Perdido Bays Estuary Program, 2022). Indeed, the predicted importance of certain grid cells in providing filtration services (Figures 3a,b and 4, Table S2) is already playing an important role, alongside other criteria, in site selection for restoration efforts (PPBEP personal comm.).

In addition to the outlined role these model outputs can and are playing in restoration goal setting and site selection, an understanding of the absolute and relative contribution to the filtration service of locations within the PBS will be used in additional forums to educate various audiences and contribute to long-term changes. For example, the outputs from the filtration model along with other critical pieces of environmental and oyster-centric data will be components that inform a NOAA Bay Watershed Education Training (B-WET) project led by PPBEP and partners, as well as other education and outreach efforts led by the PPBEP.

In conclusion, by coupling an oyster habitat suitability map with a linked hydrodynamic-oyster filtration model for the PBS, it is possible to highlight the areas within the bay that are suitable for oyster habitat restoration, and which will yield the greatest increase in filtration services. Such information is proving practically useful in terms of restoration planning and management, site selection, and for stakeholder engagement and outreach and education programs. The potential to inform decision making based on a stakeholder identified ecosystem service of importance, is a powerful tool to increase stakeholder buy-in and engagement, inform funding or granting agencies, and to increase the potential return on investment.

## Supplementary Material

Supplement1

## Figures and Tables

**TABLE 1:** Overview of variables known to impact oyster clearance rates (filtration). Adapted from Zu Ermgassen et al. (2013), and availability of data from the PBS

Variable	Effect on clearance rate	Function	Reference	Variable available for PBS?
Temperature	Unimodal with optimum filtration at ~27°C	$\text{CR}={\text{CR}}_{\max}e^{-0.015{\left(T-27\right)}^{2}}$	Newell and Langdon (1996)	Yes, spatially explicit from hydrodynamic model
Salinity	Steep decline below 7.5 ppt	CR(S)=0.5(1+tanh(S−7.5))	Cerco and Noel (2005)	Yes, spatially explicit from hydrodynamic model
Dissolved oxygen	Strong decrease <2 mg L	$\text{CR}(\text{DO})=\frac{1}{1+e\left(1.1\frac{1-\text{DO}}{1-0.7}\right)}$	Cerco and Noel (2005)	Effects on CR are negligible >2 mg L^−1^ (Cerco & Noel, 2005). Habitat suitability model excludes areas where DO <2 mgL^−1^ areas not suitable for oysters
Particle Size	Retain particles >5 μm at high efficiency	NA	Riisgaard (1988)	Not available
Seston concentration	CR low at low (<5 mg L^−1^) and high (>25 mg L^−1^) TSS	$f(\text{TSS})=0.1\left(\text{TSS}<5\;{\text{mgL}}^{-1}\right)$ $=1\left(5\leq \text{TSS}\leq 25\;{\text{mgL}}^{-1}\right)$ $=0.2\left(25<\text{TSS}\leq 100\;{\text{mgL}}^{-1}\right)$ $=0\left(\text{TSS}>100\;{\text{mgL}}^{-1}\right)$	Cerco and Noel (2005)	Not available
Flow rate	Effect poorly understood	NA	Newell and Langdon (1996), Harsh and Luckenbach (1999)	Not available
Oyster size	CR increases with oyster dry mass, which in turn increases in relation to length by an exponent of 0.58	$\text{DTW}={\text{L}}^{0.58}$	Cranford et al. (2011)	Three size classes modeled as density data available for both market and submarket size oysters

**TABLE 2:** Final oyster parameters used in the Pensacola Bay oyster filtration model under a “successful” restoration scenario.

Oyster size class	Mean SH (mm)	Reference	Mean density m^−2^	Reference
Mean < 25 mm	16	La Peyre, Humphries, et al. (2014)	61	La Peyre, Furlong, et al. (2014)
Mean 25–75 mm	56	Nevins et al. (2014)	125	La Peyre, Furlong, et al. (2014)
Mean > 75 mm	98	Nevins et al. (2014)	34	La Peyre, Furlong, et al. (2014)

## Data Availability

Results from the hydrological model will be made accessible through the EPA Environmental Dataset Gateway. Results from the filtration model are provided in the supplementary materials.
