# Supplementary material for: Using an ecosystem service model to inform restoration planning: A spatially explicit oyster filtration model for Pensacola Bay, Florida
Source: Conserv Sci Pract. Author manuscript; Available in PMC 2025 Jan 10. (PMC11457018; doi:10.1111/csp2.13061)
Supplement: Supplement1 [file NIHMS1987905-supplement-Supplement1.docx]

Supporting Information

**Hydrodynamic Model-Data Summary**

Data used in model-data comparisons focus principally on three continuous water quality monitoring instruments deployed in Escambia Bay, in the west-central PBS (Figure S1). Instruments were deployed at the surface and bottom of the mid-bay station P5 and at the bottom of station P2 in 2014. Instruments were deployed at the surface and bottom of P5 and at the bottom of P5M and P5E in 2016. Model calibration was performed using a one-year simulation in 2016, and further validation was performed using a one-year simulation in 2014. The 2014 and 2016 calibration/validation years represent years with relatively low and high spring discharge, respectively, and were selected to evaluate the model’s accuracy in addressing varying flow conditions with a single calibration. Summaries of water surface elevations, temperature, and salinity are provided in the appendix (Figures S2-5).


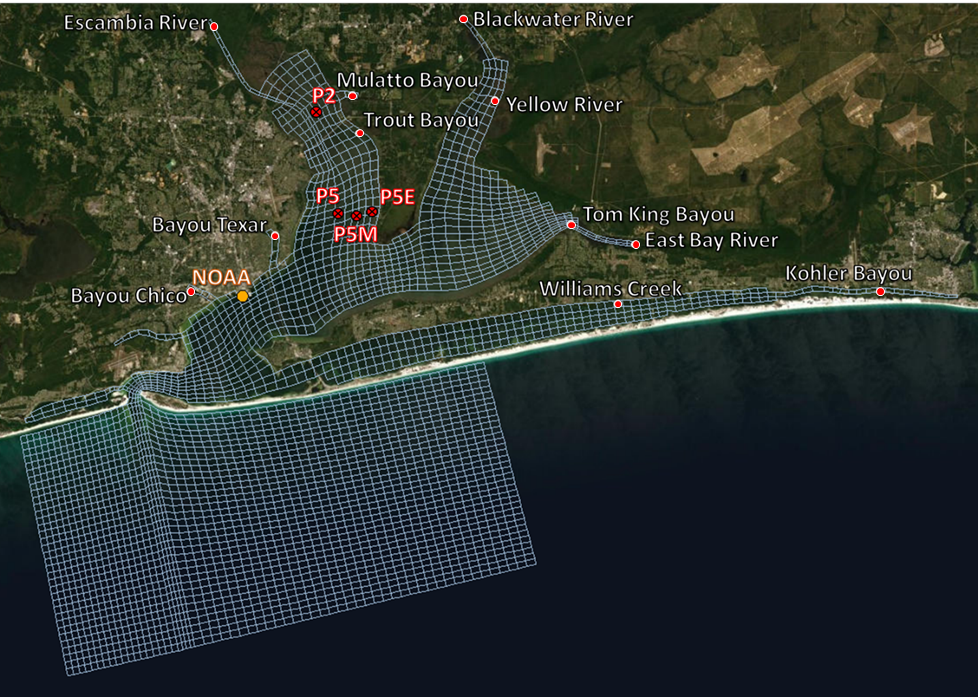
Figure S1. Pensacola Bay System EFDC Model Domain. Red crossed circles indicate continuous water quality monitoring stations used for calibration/validation. The orange circle indicates the NOAA station used for wind and tidal boundary forcing. Red filled circles indicate freshwater discharge locations.


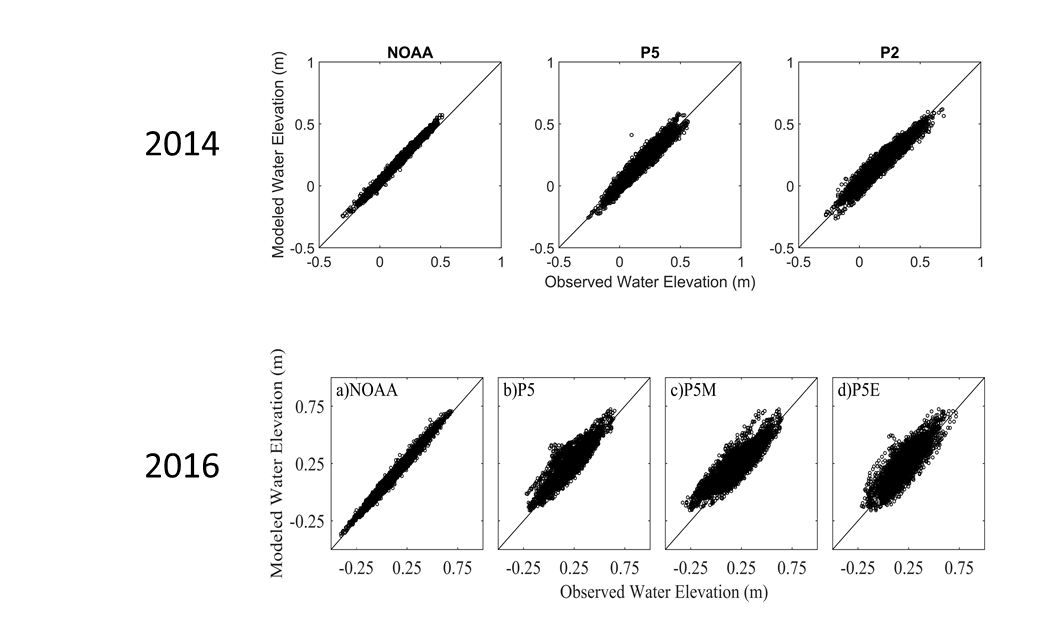


Figure S2. Modeled versus observed water surface elevation (m) relative to mean sea level (MSL) for 2014 and 2016.


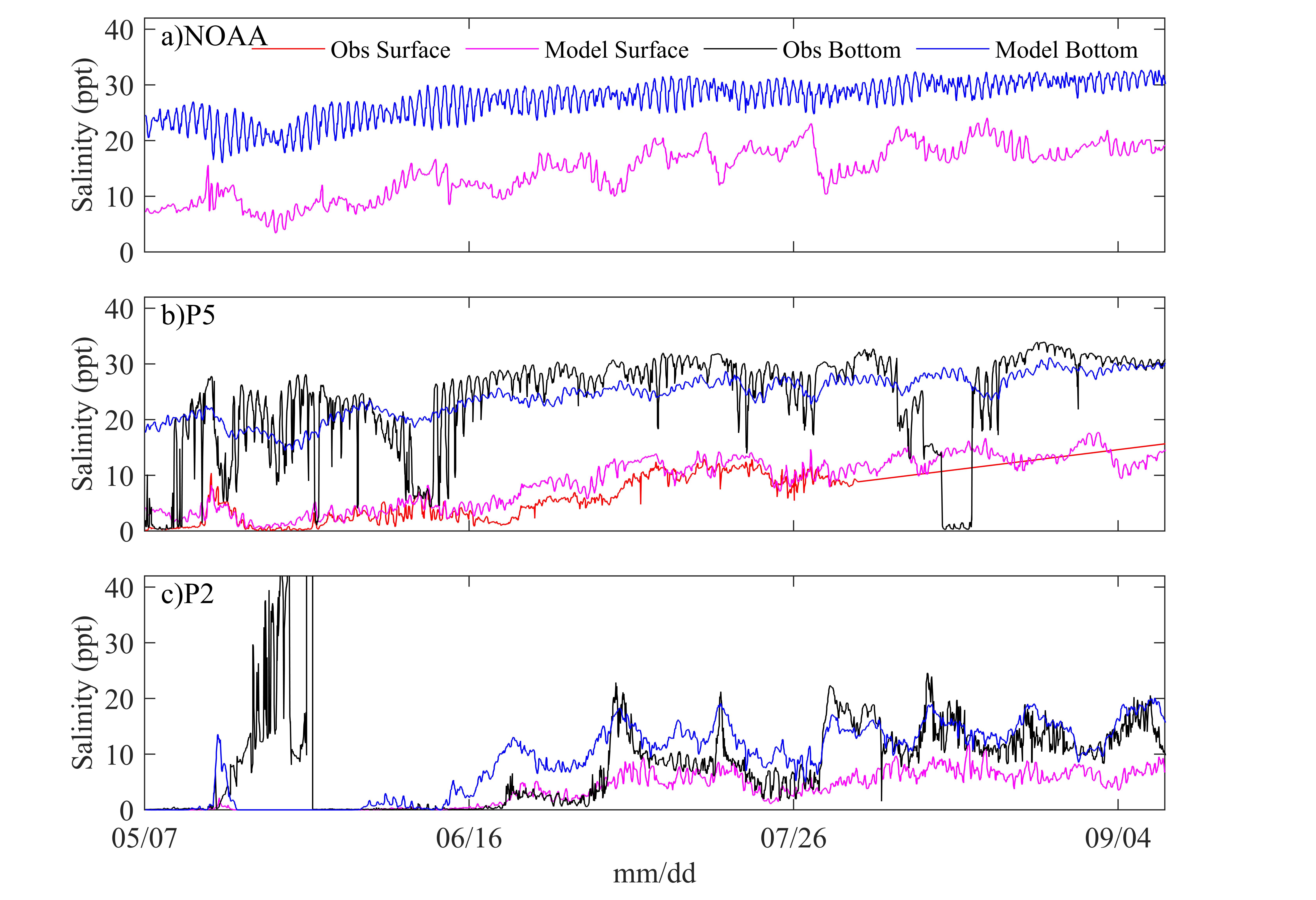


Figure S3. Modeled and observed salinity at each station in 2014.


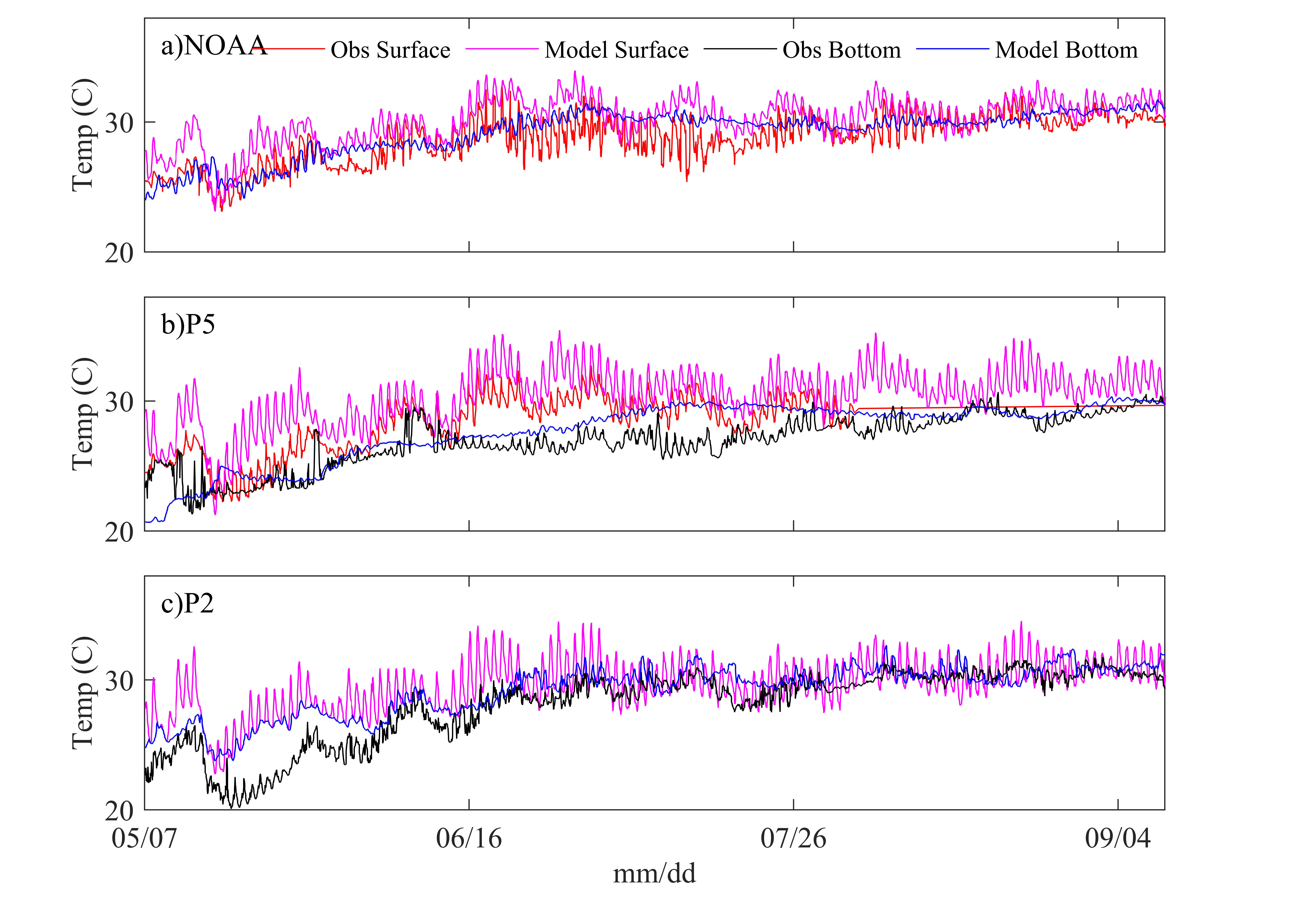


Figure S4 Modeled and observed temperature (C) at each station in 2014.

**
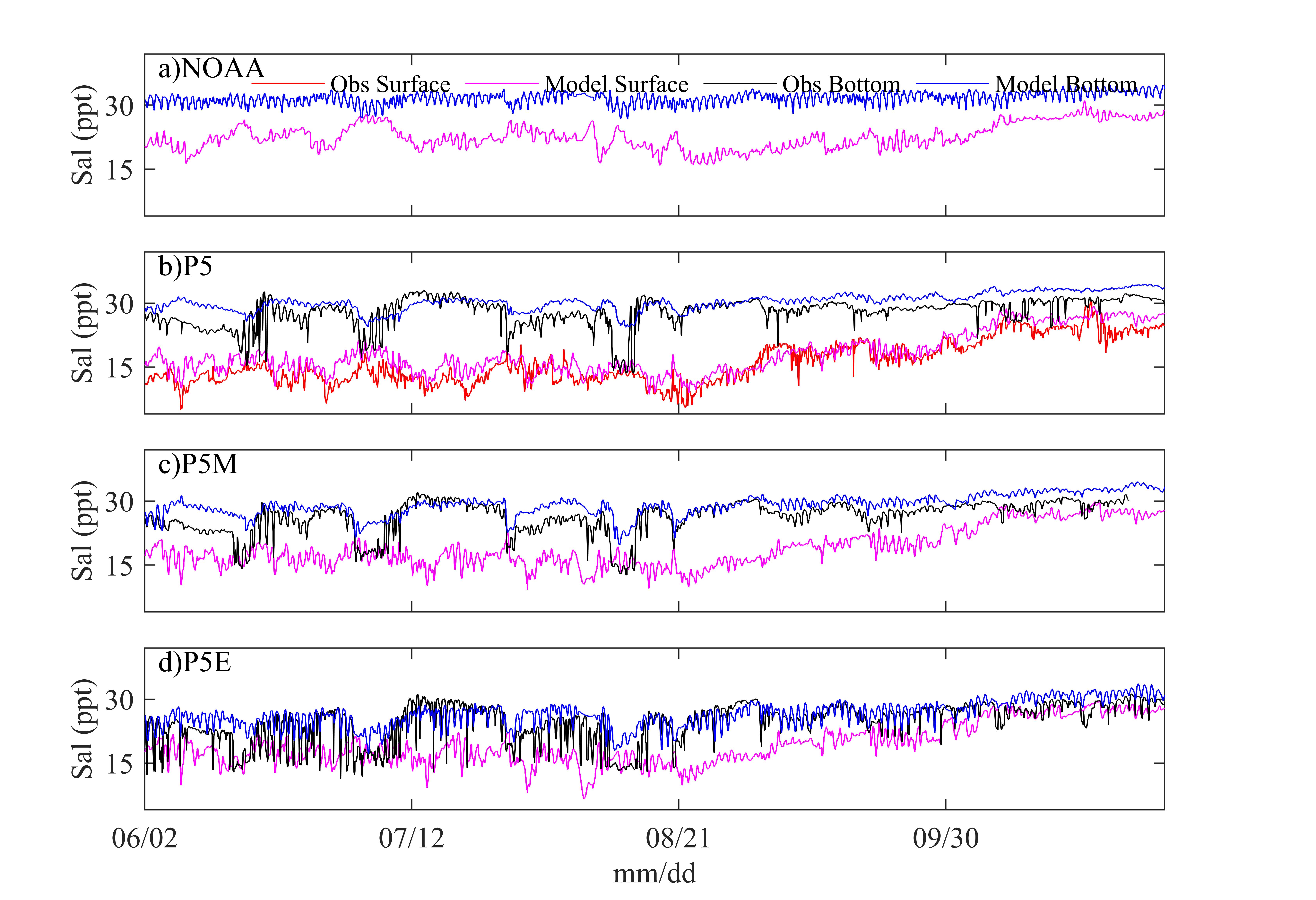
**

Figure S5. Modeled and observed salinity at each station in 2016.

**
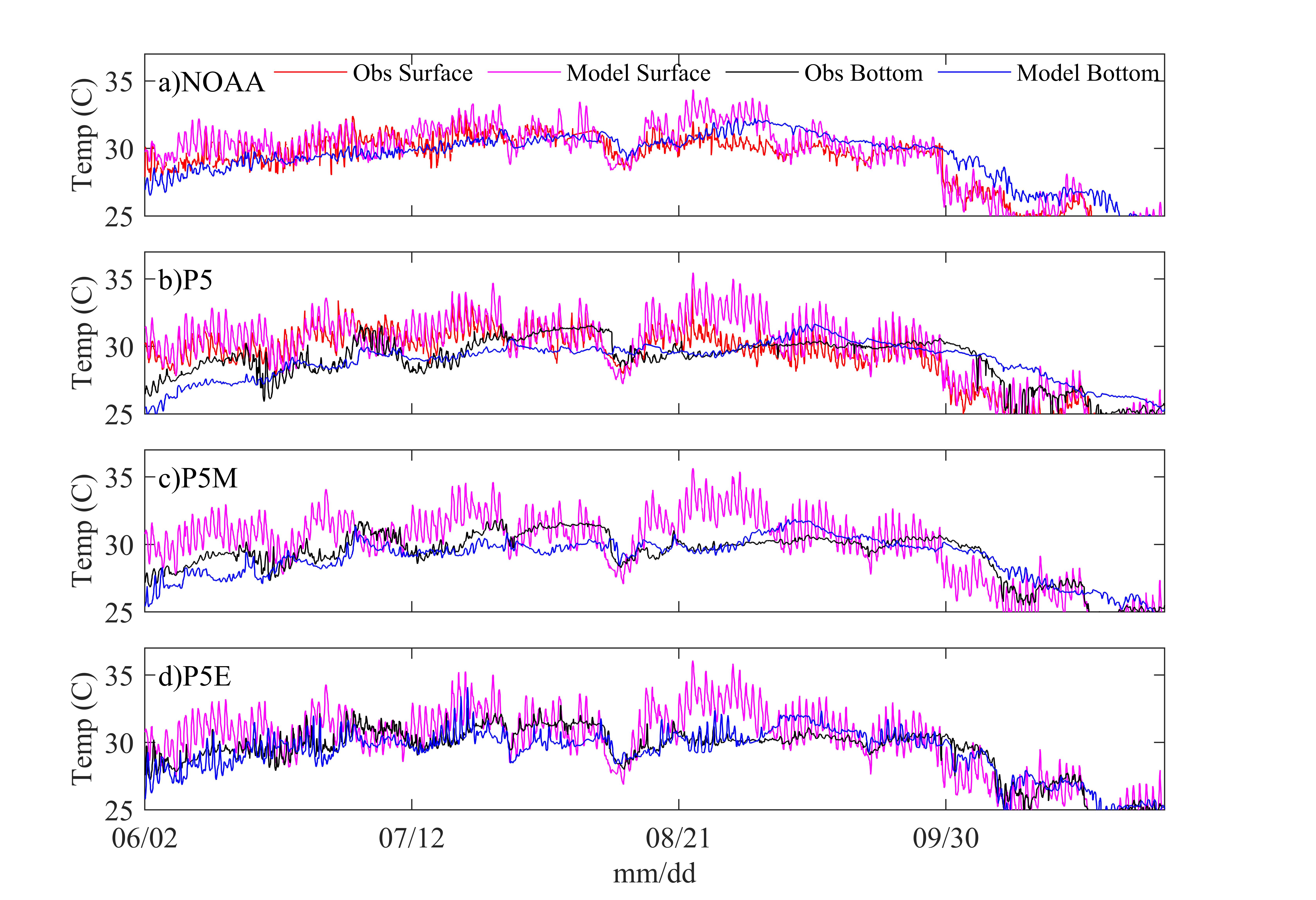
**

Figure S6. Modeled and observed temperature (C) at each station in 2016.

**Summary of Pensacola Bay Oyster Habitat Suitability Model Components**

The purpose of this supporting information is to provide further details regarding the development of the Habitat Suitability Model described in full in Geselbracht et al*. in review.*

Table S1. Pensacola Bay Oyster Habitat Suitability Model Components and Scoring. From Geselbracht et al. *in review*.

| Component | Factor | Reference | Model Scoring |
| --- | --- | --- | --- |
| Biological,  Chemical  And Physical | Present-Day Oyster Beds (2009) | FWRI | Reefs Present = 1  Reefs Absent = 0 |
|  | Historical Oyster Beds (1883) | US Fish Commission | Reefs Present = 1  Reefs Absent = 0 |
|  | Dissolved Oxygen | EPA | DO Conc. < 2 mg/l = 0  DO Conc. ≥ 2 mg/l = 1 |
|  | Seagrass | FWRI | Seagrass Present = 0  Seagrass Absent = 1 |
|  | Sediments | EPA | Mud = 0  Muddy Sand = 0.25  Sand = 0.5 |
|  | Salinity | EPA | S < 5 = 0.5  S ≥ 5 = 1.0 |
|  | Recruitment | Arnold et al. (2017) | variable from 0 -1 |
| Avoidances | Aquaculture and Shellfish Lease Areas in the Study Area | FDACS | N/A |
|  | Navigation channels | NOAA | N/A |


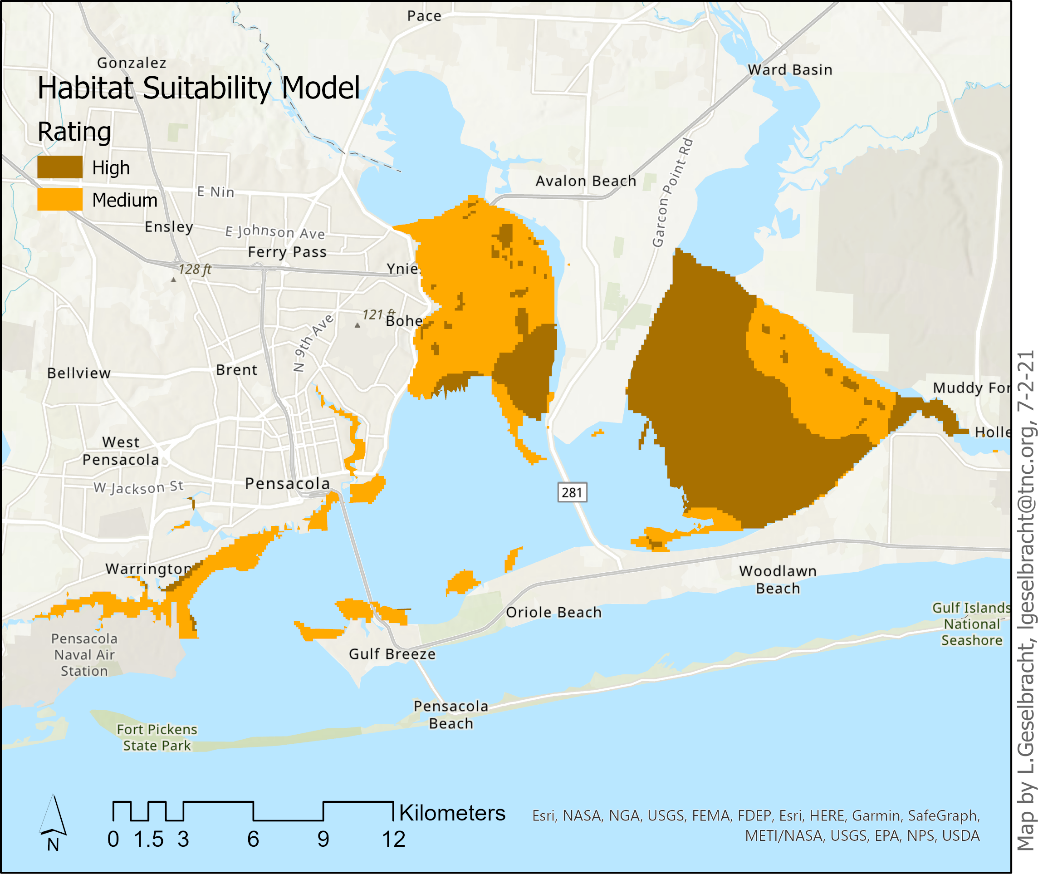


Figure S7. Areas of the Pensacola Bay System which were classified as either high or medium habitat suitability. From Geselbracht et al. *in review.*

**Results from the oyster filtration service model**

Table S2. Cell ID, filtration contribution, average filtration rate per cell and per m^2^ for the fifty topped ranked cells. The location of cells within the PBS can be determined from Figure 4 in the main manuscript.

| Cell ID | Restoration order | Area restored in cell (ha) | Filtration contribution | Average FR for whole cell (m^3^ h^-1^) | Filtration per m^2^oyster (L h^-1^) |
| --- | --- | --- | --- | --- | --- |
| 2342 | 1 | 2.3 | 0.018083 | 6119 | 264 |
| 2327 | 2 | 3.6 | 0.014848 | 10118 | 283 |
| 2386 | 3 | 2.0 | 0.020457 | 6129 | 300 |
| 2293 | 4 | 3.8 | 0.009057 | 10356 | 272 |
| 2248 | 5 | 5.0 | 0.005554 | 13437 | 268 |
| 2294 | 6 | 3.2 | 0.011029 | 10739 | 334 |
| 2891 | 7 | 13.3 | 0.008097 | 30160 | 228 |
| 2313 | 8 | 3.7 | 0.011602 | 12588 | 344 |
| 2263 | 9 | 4.2 | 0.006379 | 12642 | 300 |
| 2222 | 10 | 3.4 | 0.006597 | 10665 | 309 |
| 2935 | 11 | 11.5 | 0.005474 | 32040 | 279 |
| 2932 | 12 | 11.0 | 0.004196 | 27833 | 254 |
| 2328 | 13 | 2.9 | 0.008359 | 10629 | 365 |
| 2890 | 14 | 10.4 | 0.005123 | 22191 | 213 |
| 2234 | 15 | 2.6 | 0.00387 | 9333 | 361 |
| 2278 | 16 | 4.2 | 0.007372 | 11861 | 282 |
| 2858 | 17 | 2.1 | 0.005009 | 3603 | 172 |
| 2966 | 18 | 11.9 | 0.004139 | 30025 | 253 |
| 2221 | 19 | 3.3 | 0.005025 | 9580 | 287 |
| 2312 | 20 | 3.8 | 0.008071 | 12959 | 343 |
| 2969 | 21 | 11.5 | 0.003739 | 32120 | 280 |
| 2560 | 22 | 5.5 | 0.007305 | 21230 | 383 |
| 2383 | 23 | 4.0 | 0.011497 | 12807 | 316 |
| 2927 | 24 | 12.0 | 0.004269 | 30020 | 250 |
| 2892 | 25 | 11.9 | 0.004881 | 27847 | 234 |
| 2968 | 26 | 11.7 | 0.00423 | 31754 | 272 |
| 2517 | 27 | 6.6 | 0.005112 | 25628 | 386 |
| 2922 | 28 | 9.4 | 0.006007 | 21455 | 229 |
| 3000 | 29 | 11.2 | 0.003457 | 28154 | 250 |
| 2971 | 30 | 11.5 | 0.00434 | 33708 | 292 |
| 2416 | 31 | 3.9 | 0.006656 | 11686 | 300 |
| 2933 | 32 | 11.8 | 0.005052 | 30448 | 259 |
| 2220 | 33 | 1.9 | 0.004452 | 4861 | 254 |
| 2558 | 34 | 5.3 | 0.004835 | 20681 | 387 |
| 2936 | 35 | 11.0 | 0.005616 | 32108 | 291 |
| 2959 | 36 | 9.6 | 0.00392 | 22077 | 231 |
| 2739 | 37 | 6.1 | 0.005771 | 18072 | 296 |
| 3049 | 38 | 8.5 | 0.003353 | 22112 | 260 |
| 2561 | 39 | 7.4 | 0.00531 | 27916 | 379 |
| 2931 | 40 | 12.1 | 0.004429 | 30245 | 251 |
| 2973 | 41 | 8.7 | 0.004547 | 26041 | 299 |
| 2374 | 42 | 1.5 | 0.005596 | 6061 | 415 |
| 2887 | 43 | 9.6 | 0.003404 | 18488 | 193 |
| 2485 | 44 | 4.7 | 0.004363 | 18282 | 392 |
| 3002 | 45 | 11.0 | 0.002812 | 29195 | 265 |
| 2997 | 46 | 10.2 | 0.00225 | 23818 | 234 |
| 2375 | 47 | 2.4 | 0.004279 | 8991 | 378 |
| 2728 | 48 | 6.6 | 0.004913 | 13903 | 210 |
| 2923 | 49 | 9.1 | 0.004744 | 20978 | 231 |
| 3044 | 50 | 7.7 | 0.004032 | 18243 | 235 |
| 2934 | 51 | 11.6 | 0.005625 | 30886 | 267 |
| 2993 | 52 | 9.0 | 0.002875 | 19908 | 221 |
| 2514 | 53 | 4.5 | 0.005121 | 17897 | 400 |
| 2314 | 54 | 1.2 | 0.003644 | 4991 | 430 |
| 2926 | 55 | 10.4 | 0.004051 | 25616 | 247 |
| 2626 | 56 | 5.6 | 0.002887 | 20711 | 369 |
| 2371 | 57 | 1.9 | 0.004589 | 6208 | 319 |
| 2967 | 58 | 11.7 | 0.003939 | 30731 | 263 |
| 3003 | 59 | 10.7 | 0.002432 | 28789 | 269 |
| 2930 | 60 | 10.9 | 0.004302 | 27317 | 250 |
| 2702 | 61 | 6.3 | 0.002453 | 21199 | 334 |
| 2484 | 62 | 3.5 | 0.004102 | 14042 | 401 |
| 2999 | 63 | 11.1 | 0.002751 | 26811 | 241 |
| 2403 | 64 | 3.3 | 0.004794 | 10887 | 328 |
| 2937 | 65 | 8.2 | 0.005927 | 24766 | 301 |
| 2730 | 66 | 6.8 | 0.003333 | 18209 | 270 |
| 2925 | 67 | 9.6 | 0.003153 | 23278 | 242 |
| 3001 | 68 | 11.1 | 0.002949 | 28341 | 256 |
| 2960 | 69 | 10.1 | 0.004511 | 23572 | 233 |
| 2889 | 70 | 9.2 | 0.003237 | 18873 | 205 |
| 3069 | 71 | 6.6 | 0.002353 | 15961 | 242 |
| 2593 | 72 | 5.7 | 0.002954 | 21582 | 378 |
| 3007 | 73 | 9.3 | 0.003495 | 27040 | 292 |
| 2249 | 74 | 0.7 | 0.001324 | 2932 | 422 |
| 3022 | 75 | 8.6 | 0.003985 | 20719 | 241 |
| 2395 | 76 | 2.0 | 0.005476 | 7171 | 354 |
| 2939 | 77 | 4.7 | 0.004733 | 14279 | 307 |
| 2958 | 78 | 9.3 | 0.003716 | 20837 | 224 |
| 2625 | 79 | 6.3 | 0.003506 | 21145 | 337 |
| 2963 | 80 | 10.6 | 0.00326 | 25501 | 241 |
| 2886 | 81 | 9.2 | 0.002875 | 17517 | 191 |
| 2482 | 82 | 4.6 | 0.004589 | 16680 | 364 |
| 2631 | 83 | 5.9 | 0.002599 | 21385 | 360 |
| 3071 | 84 | 6.8 | 0.002177 | 17266 | 253 |
| Total | NA | 599.6 | 0.437 | NA | NA |

**Results from modelled “highly successful” oyster restoration**

In addition to the results presented in the main body of the paper, the filtration services were also determined for a “highly successful” oyster restoration scenario, which was based on field derived oyster densities for sites where >50 oysters m^-2^, as reported in La Peyre et al. 2014 (Table S3).

Table S3 Oyster parameters used in PBS oyster CR model- Scenario “Highly successful” restoration.

| Oyster size class | Mean SH (mm) | Reference | Mean density m^-2^ | Reference |
| --- | --- | --- | --- | --- |
| Mean <25mm | 16 | La Peyre et al. 2014b | 431 | La Peyre et al. 2014a |
| Mean 25-75mm | 56 | Nevin et al. 2014 | 227 | La Peyre et al. 2014a |
| Mean >75mm | 98 | Nevin et al. 2014 | 80 | La Peyre et al. 2014a |

Figure S8

The predicted fraction of particles in the hydrodynamic model which are cleared from the water column over time once 17.7% of high habitat suitability and 11% of medium habitat suitability area is restored under a “highly successful” restoration scenario (50 oysters m^-2^). “Full estuary filtration” is achieved in ~14 days.


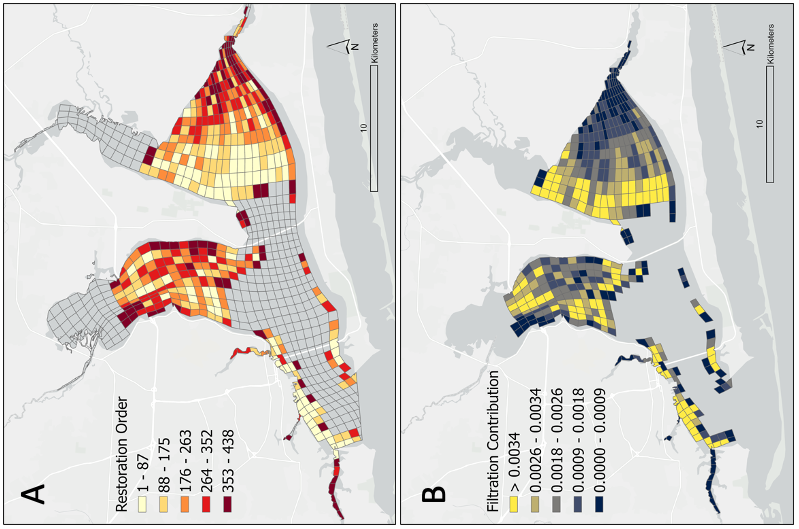


Figure S9

Maps illustrating a. restoration order (top), and b. filtration contribution of cells (bottom) under a “highly successful” restoration scenario, two-week model run.


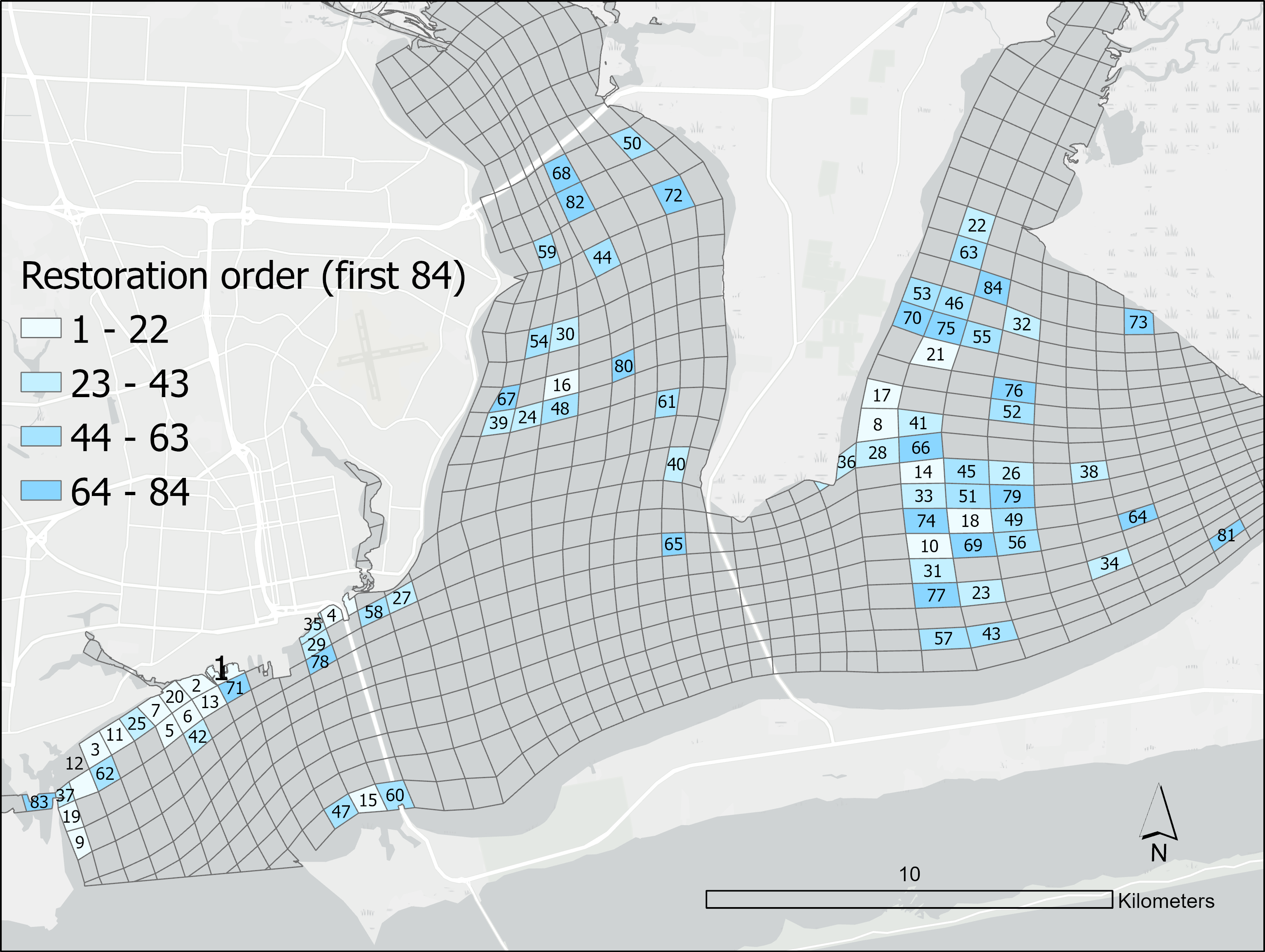


Figure S10 Restoration order of the first 84 cells under “highly successful” restoration scenario, which would provide the greatest additional filtration if restored sequentially, starting with grid cell 1. The 84 cells represent ~600 ha of potential restored oyster reef area. This area is equivalent to that agreed by stakeholders as an initial oyster reef restoration goal (Goal 5 in PPBEP 2022).

**References**

Geselbracht L, Johnston M, DeAngelis B, Birch A. *In review*. Stakeholder informed adaptive habitat suitability model for the eastern oyster (*Crassostrea virginica*) in the Pensacola Bay System, Florida, USA.

La Peyre, M., Furlong, J., Brown, L.A., Piazza, B.P., Brown, K., 2014a. Oyster reef restoration in the northern Gulf of Mexico: Extent, methods and outcomes. *Ocean & Coastal Management* 89, 20-28.

La Peyre, M.K., Humphries, A.T., Casas, S.M., La Peyre, J.F., 2014b. Temporal variation in development of ecosystem services from oyster reef restoration. *Ecological Engineering* 63, 34-44.

Nevins, J.A., Pollack, J.B., Stunz, G.W. 2014. Characterizing nekton use of the largest unfished oyster reef in the United States compared with adjacent estuarine habitats. *Journal of Shellfish Research* 33, 227-238.

Oyster Metrics Workgroup, 2011. Restoration goals, quantiative metrics and assessment protocols for evaluating success on restored oyster reef sanctuaries. Submitted to the Sustainable Fisheries Goal Implementation Team for the Chesapeake Bay Program, p. 32.

Pensacola & Perdido Bays Estuary Program (PPBEB), 2022. Comprehensive Conservation and Management Plan: A Prescription for healthy bays. Pensacola & Perdido Bays Estuary Program p.21
